# Supplementary material for: Engineering artificial photosynthesis based on rhodopsin for CO2 fixation
Source: Nat Commun. 2023 Dec 4;14:8012. doi: 10.1038/s41467-023-43524-4 (PMC10696030; doi:10.1038/s41467-023-43524-4)
Supplement: Supplementary file 1 — Supplementary Information [file 41467_2023_43524_MOESM1_ESM.pdf]

Engineering artificial photosynthesis based on rhodopsin for CO_2 fixation

Tu *et al.*

Supplementary Method 1. Heme staining of SDS-PAGE

Whole-cell proteins were extracted by FastPrep-24™ Classic Bead Beating Grinder and Lysis System (MP biomedicals). Cell extracts were suspended in lithium dodecyl sulfate. The samples were run in NuPAGE™ 10% Bis-Tris Gel (Invitrogen) at 16 °C. Electrophoresis was initiated at 100 V for 30 minutes and then increased to 180 V for an additional 60 minutes. The 3,3′,5,5′-tetramethylbenzidene (TMBZ) peroxidase stain method was adapted from Thomas et al [1] to identify cytochromes *c*, as reported by Jensen et al [2]. TMBZ was dissolved in methanol to 6.3 mM and mixed in a 3:7 ratio solution with TMBZ solution to 0.25 M Sodium acetate (pH=5). The gel was immersed in the mixture in the dark with periodic mixing for 2 h. Hydrogen peroxide was added to reach a final concentration of 30 mM, and the heme-containing protein bands were visualized 30 min after peroxide addition.

Supplementary Method 2. Proton pumping assay

R. eutropha RHM5-GR-Mtr strains were precultured in TSB medium at 30 °C and induced by 0.1% arabinose and 5 μg mL^{-1} retinal, with and without the addition of canthaxanthin, respectively. Then we harvested cells, washed them with unbuffed solution (10 mM NaCl, 10 mM $MgSO_4 \cdot 7H_2O$, and 0.1 mM $CaCl_2$) and resuspended them in the unbuffered solution to obtain an OD_{600} of ~1.5. The cell suspension was placed in the dark until the pH was stabilized, which was measured by Orion Star™ A211 Benchtop pH Meter (ThermoFisher, UK). Following stabilization, the suspension was exposed to illumination with an intensity of ~150 μmol m^{-2} s^{-1} for 1 minute, and the ΔpH was calculated.

Supplementary Method 3. Comparison of cell viability with and without canthaxanthin

R. eutropha RHM5-GR-Mtr strains were precultured as above, with and without the addition of canthaxanthin, respectively. Subsequently, cells were harvested, washed with phosphate buffer solution (PBS) twice and resuspended in PBS to an OD of 0.6. The cell suspensions were incubated at 30 °C for 120 hours under illumination with an intensity of ~150 μmol/s/m^2, and 100 μL of each dilution was spread on LB agar plates to obtain the Colony-forming unit (CFU) numbers.

Supplementary Table 1. Strains and plasmids used in this study.

Strains	Comments
E. coli DH5α	Commercially obtained (NEW England, UK)
E. coli S17	Commercially obtained (NEW England, UK)
E. coli MG1655	Commercially obtained (NEW England, UK)
Shewanella oneidensis MR-1	Wild-type strain
Ralstonia eutropha H16 (ATCC 17699)	Wild-type strain
Ralstonia eutropha H16 Δ*pha* (RHM5)	Wild-type strain with *phaCAB* operon encoding genes required for conversion of acetyl-CoA to polyhydroxybutyrate (PHB) deleted (gift from Min-Kyu Oh, Korea University, S. Korea).
R. eutropha-Mtr	*Ralstonia eutropha* H16: pLO11a-Mtr
R. eutropha-GR	*Ralstonia eutropha* H16: pLO11a-GR
R. eutropha-GR-Mtr	*Ralstonia eutropha* H16: pLO11a-GR-Mtr
RHM5-*can*	*Ralstonia eutropha* H16 Δ*pha*: pLO11a-*can*
RHM5-GR	*Ralstonia eutropha* H16 Δ*pha*: pLO11a-GR
RHM5--GR-Mtr	*Ralstonia eutropha* H16 Δ*pha*: pLO11a-GR-Mtr
RHM5-GR-Mtr-*can*	*Ralstonia eutropha* H16 Δ*pha*: pLO11a-GR-Mtr-*can*
Plasmids	**Comments**
pLO11a (Tc^r, RK2 *ori*, Mob^+)	Expression vector for use in *R. eutropha* with P_{BAD} promoter and downstream cloning sites (gift from Oliver Lenz, Technische Universität Berlin, Germany).
pLO11a-GR [3]	pLO11a containing the gene for GR rhodopsin from *Gloeobacter violaceus* PCC7421
pLO11a-blhDxrCRT-GR [3]	pLO11a containing *GR* gene and the retinal synthetic gene cluster
pLO11a-Mtr	pLO11a containing *MtrCAB* gene cluster from *Shewanella oneidensis* MR-1
pLO11a-GR-Mtr	pLO11a containing *GR* gene from *Gloeobacter violaceus* PCC7421 and *MtrCAB* gene cluster from *Shewanella oneidensis* MR-1
pLO11a-*can*	pLO11a containing *can* gene from *Ralstonia eutropha* H16
pLO11a-GR-Mtr-*can*	pLO11a containing *GR* gene from *Gloeobacter violaceus* PCC7421, *MtrCAB* gene cluster from *Shewanella oneidensis* MR-1 and *can* gene from *Ralstonia eutropha* H16

Supplementary Table 2. Primers used in this study.

Primer name	Sequence 5’ to 3’
pLO11a-GR1-rv	GCGTTCATCATGGGGTCTCCTCCTTAGCTAG
pLO11a-GR1-fw	CAAACTCTAACGGCCGCAGATCTGC
mtr-rv	AGGAGACCCCATGATGAACGCACAAAAATCAAAAATCG
mtr-fw	TCTGCGGCCGTTAGAGTTTGTAACTCATGCTCAGCATCAG
pLO11a-GR2-rv	CGTCAGTCATGGGGTCTCCTCCTTAGCTAG
pLO11a-GR2-fw	GATCCGCTGACGGCCGCAGATCTGC
can1-rv	ATCTGCGGCCGTCAGCGGATCGACGC
can1-fw	AGGAGACCCCATGACTGACGCCATCGCC
pLO11a-GR3-rv	CGACCCTCCTTCTAGGAGATAAGACTGCCTCCCG
pLO11a-GR3-fw	GATCCGCTGACGGCCGCAGATCTGC
can2-rv	ATCTGCGGCCGTCAGCGGATCGACGC
can2-fw	TTATCTCCTAGAAGGAGGGTCGCATGACTG
pLO11a-Mtr-rv	GAGTGTTGTTCCAGTTTGGA
pLO11a-Mtr-fw	TTATGACAACTTGACGGCTA
pLO11a-blhDxrCRT-GR-rv	TAGCCGTCAAGTTGTCATAA
pLO11a-blhDxrCRT-GR-fw	TCCAAACTGGAACAACACTC

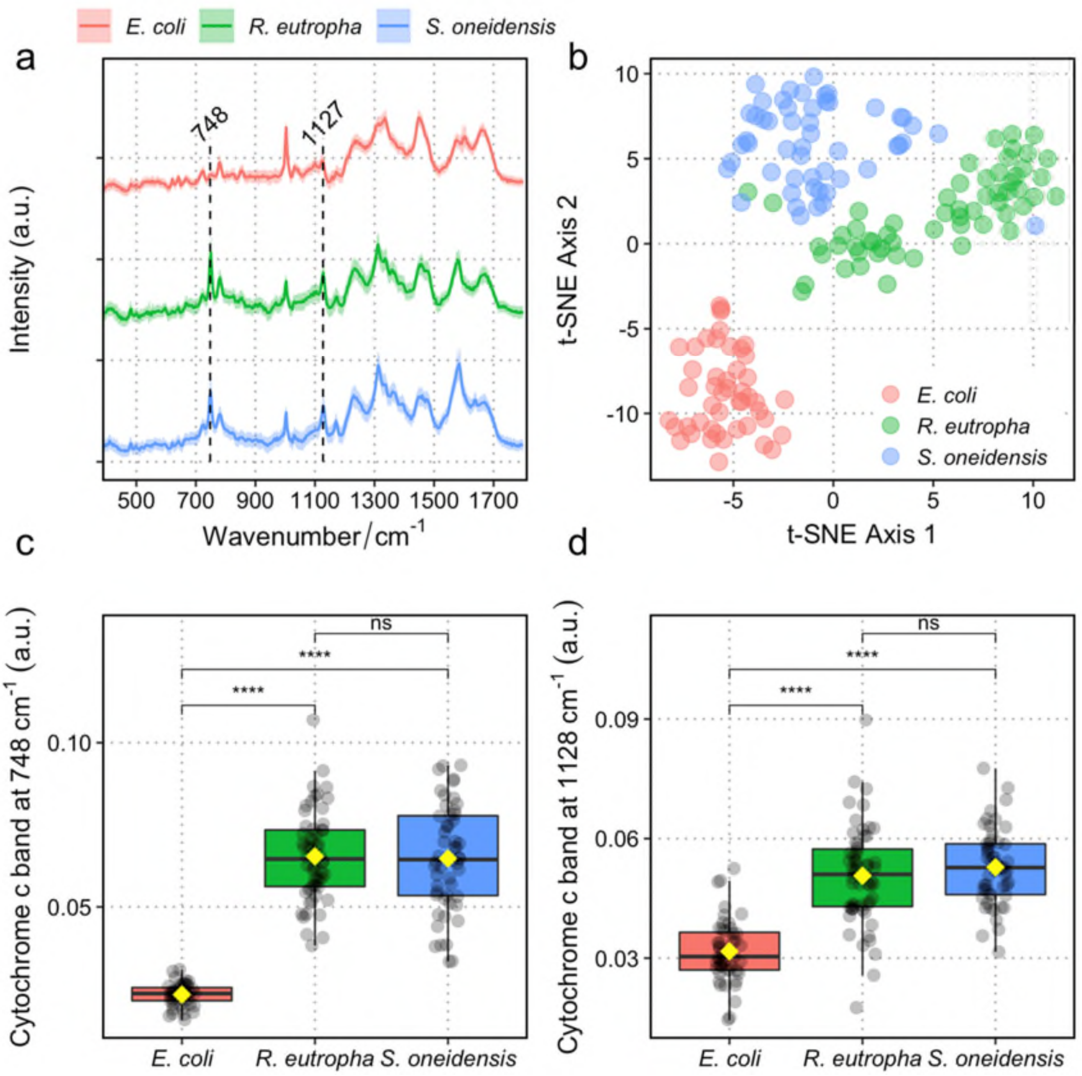

Supplementary Fig. 1. Comparison of average Raman profiles of *E. coli MG*-1655, *R. eutropha* H16 and *S. oneidensis* MR-1. (a) Average single-cell Raman spectrum of *E. coli MG*-1655, *R. eutropha* H16 and *S. oneidensis* MR-1. (b) t-SNE visualization of sing-cell Raman spectrum showing clusters of different strains. (c-d) Semi-quantification of cytochromes levels by integrating Raman bands at (c) 748 and (d) 1128 cm^{-1}. Statistics were performed with two-sided Student's *t*-test (****: $p < 0.0001$). Source data are provided as a Source Data file.

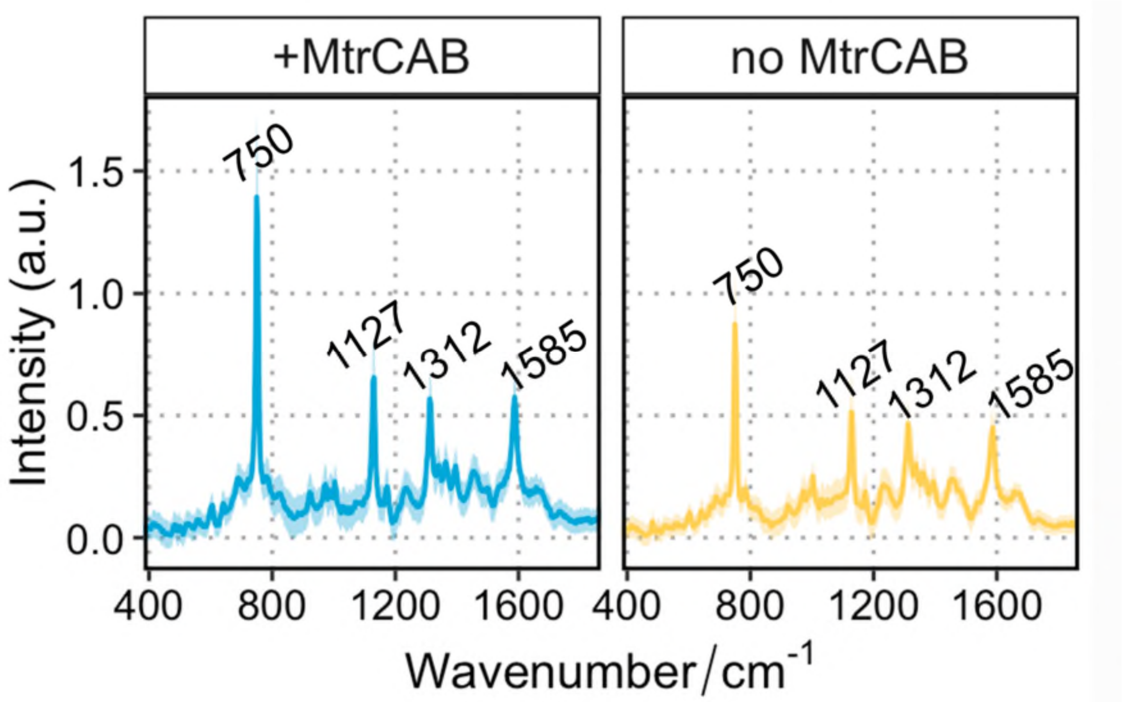

Supplementary Fig. 2. Comparison of induced and uninduced *R. eutropha*-Mtr. Average single-cell Raman spectrum of induced and uninduced strains. Four Raman bands associated with cytochrome *c* are highlighted at 748, 1128, 1312, and 1585 cm^{-1}. Source data are provided as a Source Data file.

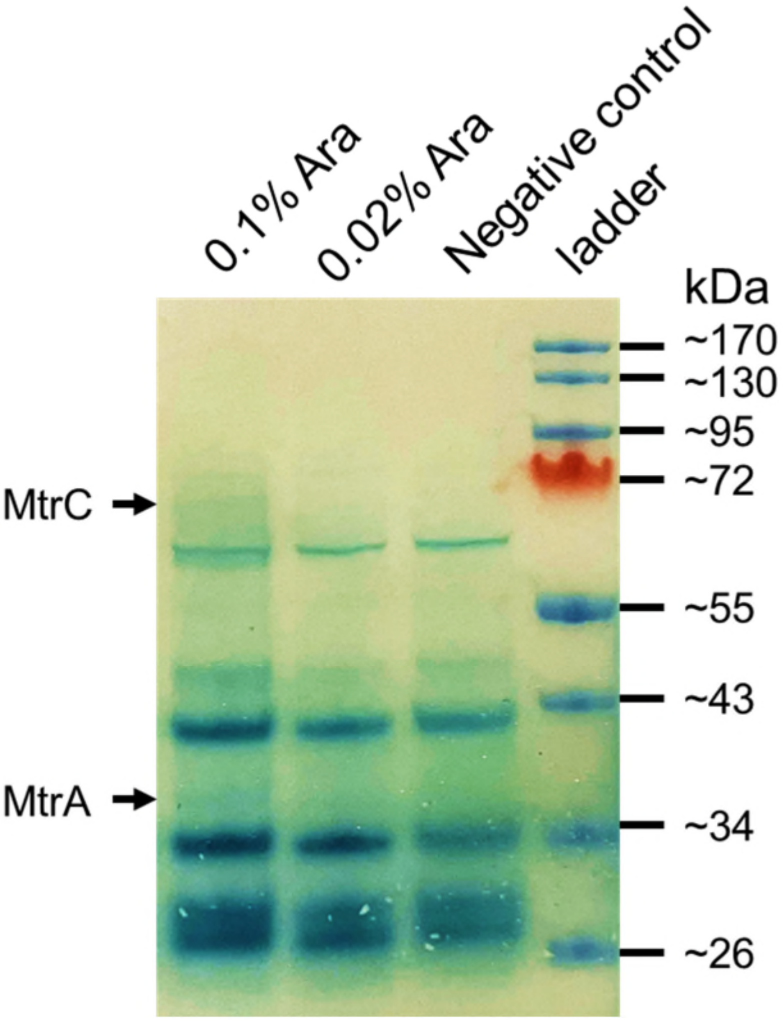

Supplementary Fig. 3. Heme staining of whole cell lysates of *R. eutropha* without expression of Mtr (as the negative control) and *R. eutropha*-Mtr at arabinose (Ara) inducer concentrations of 0.02% and 0.1%. Source data are provided as a Source Data file.

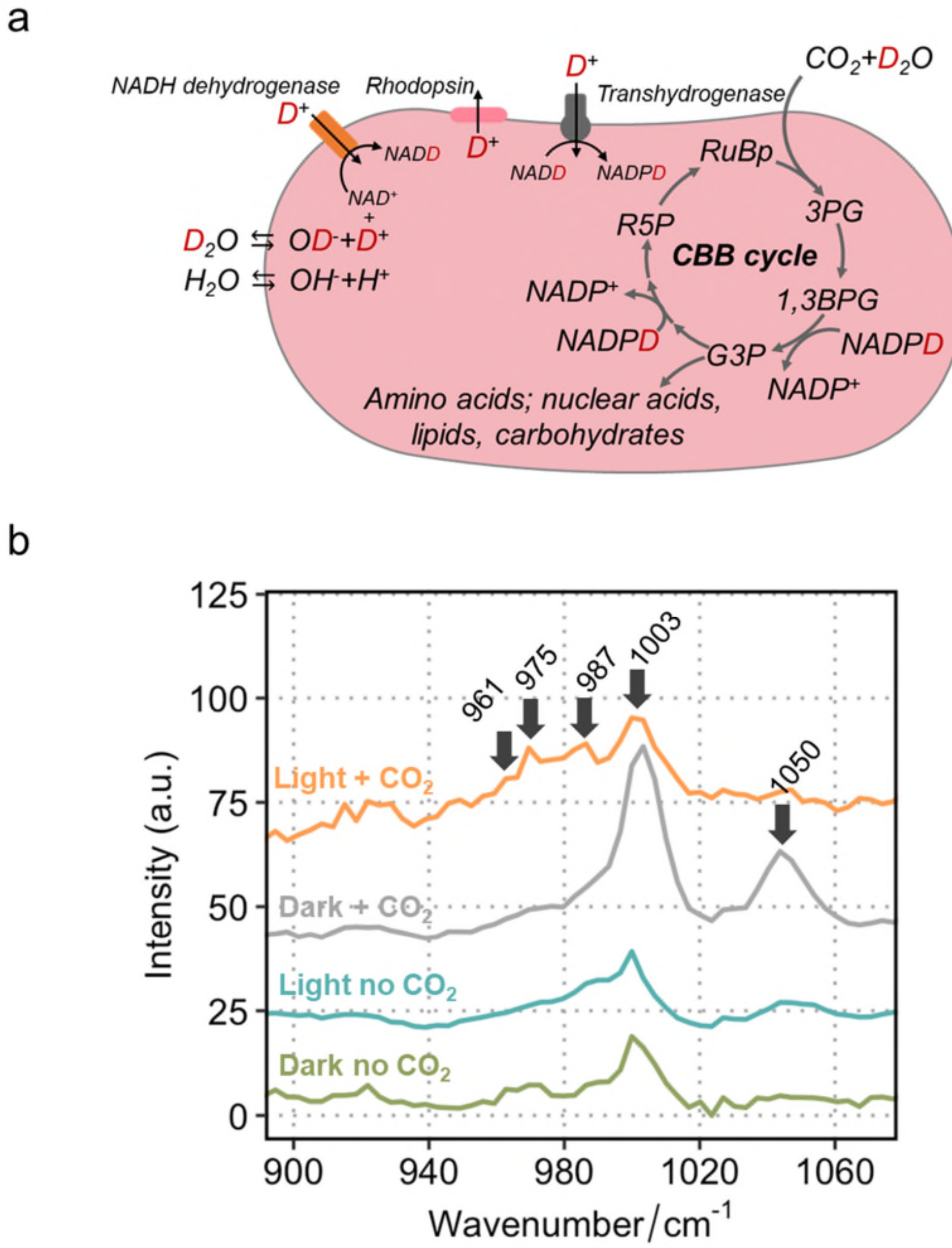

Supplementary Fig. 4. Schematic illustration of heavy water (D_2O) involved in metabolisms in CO_2 fixation (a) In the presence of D_2O, bacterial metabolism can substitute H with deuterium (D highlighted in red) to synthesise NADD and NADPD for biosynthesis. The process facilitates the assimilation of carbon source (e.g., CO_2) and formation of carbon-deuterium (C–D) bonds in biomolecules (e.g., amino acids). This D–H replacement will lead to observable shifts in the Raman spectra of cells. (b) Averaged single-cell Raman spectra of *R. eutropha*-GR-Mtr cultured with 30% D_2O under different conditions. Raman shifts were observed from 1003 cm^{-1} (Raman characteristic marker of phenylalanine) to 987, 975 and 961 cm^{-1} under light and CO_2. In contrast, bicarbonate (Raman characteristic band at 1050 cm^{-1}) remained unused within the cells in dark condition. Source data are provided as a Source Data file.

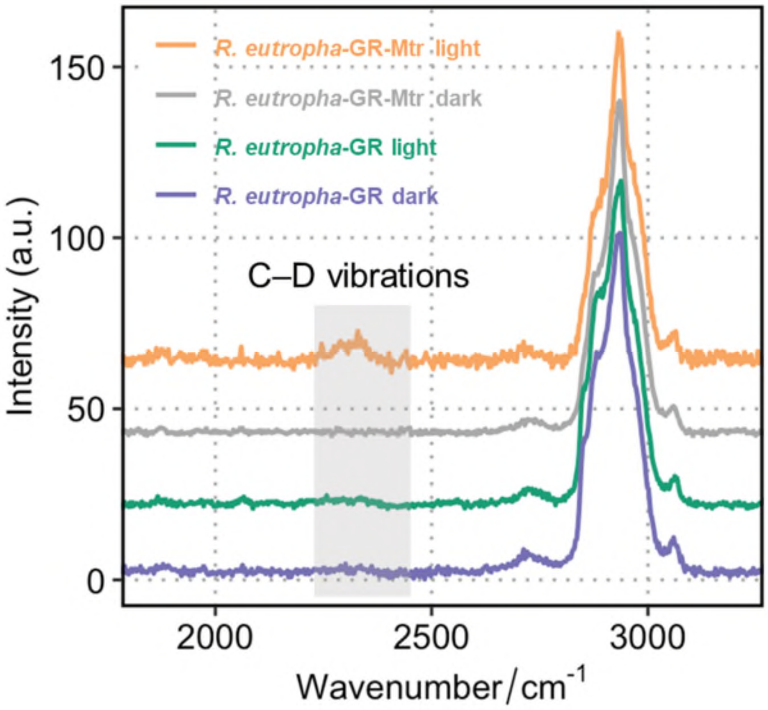

Supplementary Fig. 5. Averaged single-cell Raman spectra of *R. eutropha*-GR-Mtr and *R. eutropha*-GR cultured with 30% D_2O and CO_2 under light and dark conditions. Source data are provided as a Source Data file.

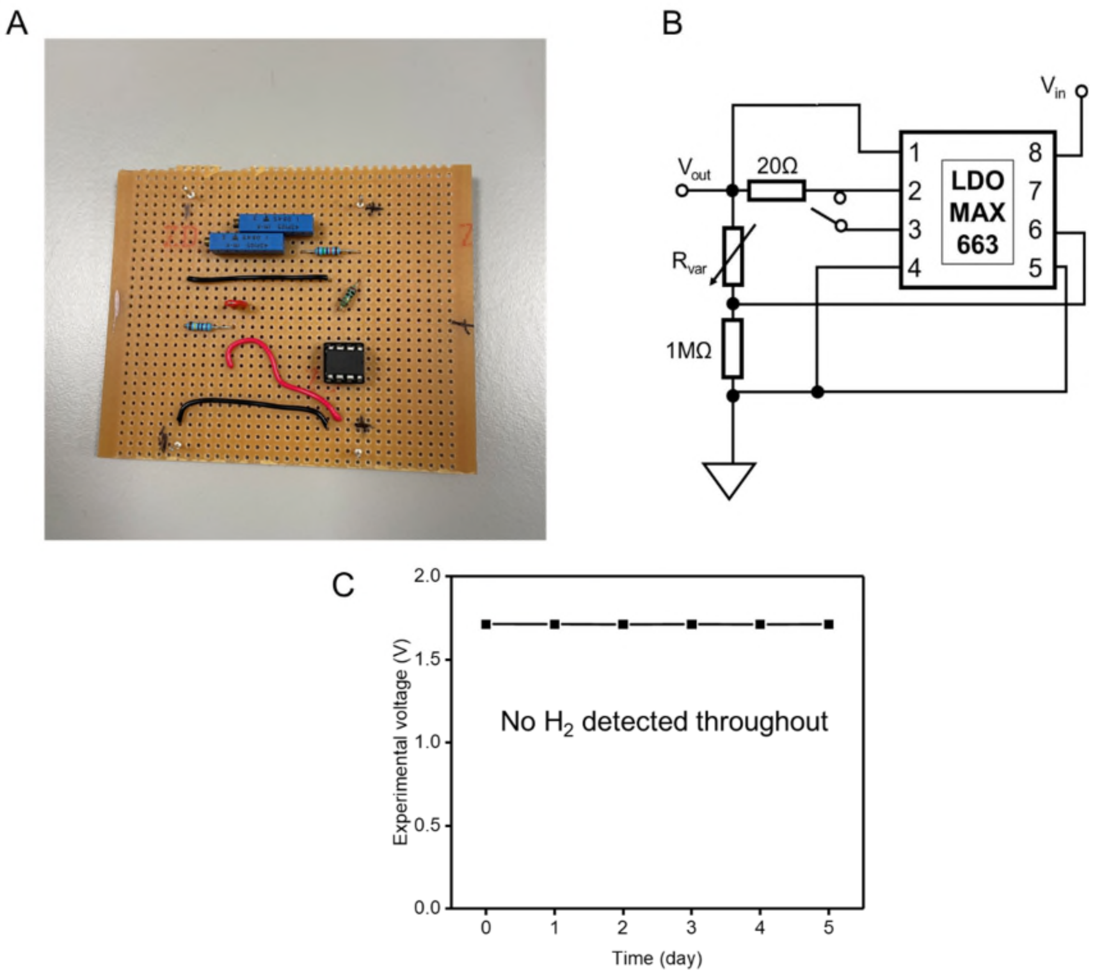

Supplementary Fig. 6. Design of a home-made voltage regulator. (a) A picture of the voltage regulator. (b) The circuit diagram of the voltage regulator (c) Volage output controlled by the voltage regulator. Source data are provided as a Source Data file.

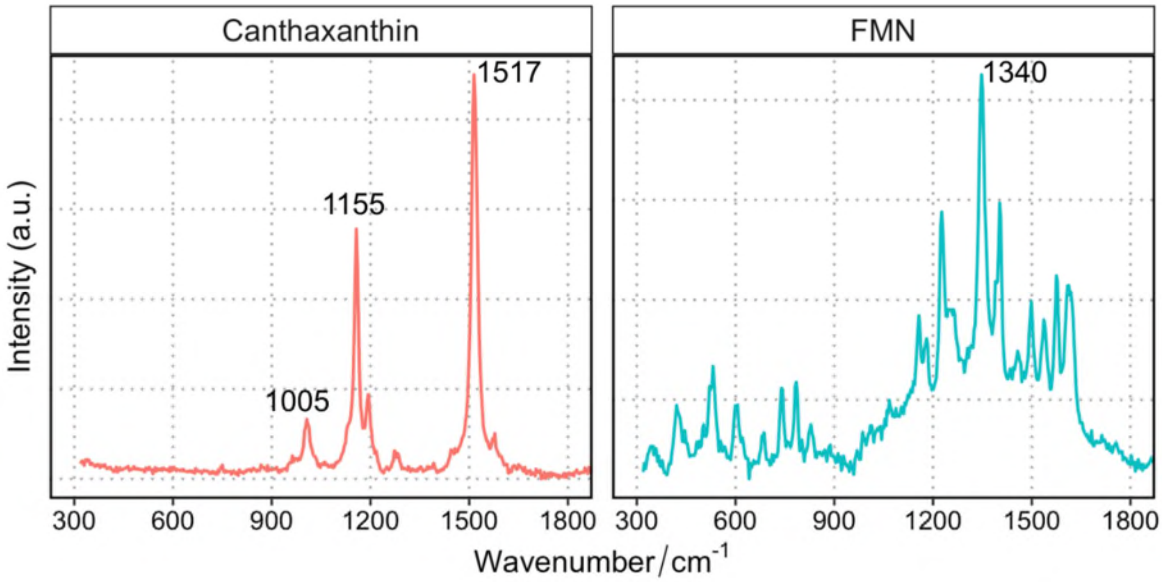

Supplementary Fig. 7. Characterization of pure canthaxanthin and flavin mononucleotide (FMN) by Raman analysis. Averaged Raman spectra of pure canthaxanthin and FMN. Source data are provided as a Source Data file.

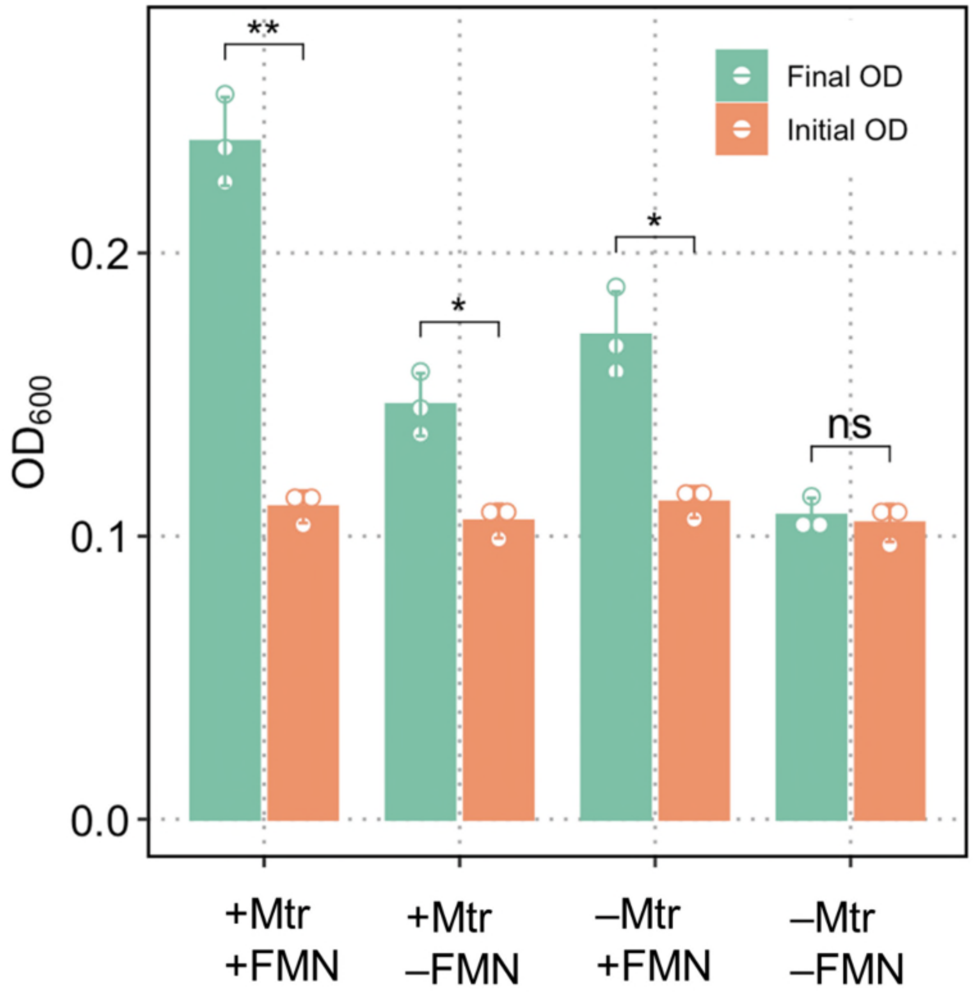

Supplementary Fig. 8. Initial and final optical density (OD_{600}) for the *R. eutropha* RHM5-GR-Mtr strains with and without inducation Mtr pathway after 5 days incubations with and without 50 µM FMN as the mediator. Statistics were performed with Student's *t*-test (**: $p < 0.01$, *: $p < 0.05$, ns: not significant). Data are means ± SD of three biological replicates. Exact *p* values are 0.00207, 0.00102, 0.0137 and 0.636 for "+Mtr, +FMN", "+Mtr, –FMN", "–Mtr, +FMN" and "–Mtr, –FMN" groups, respectively. Source data are provided as a Source Data file.

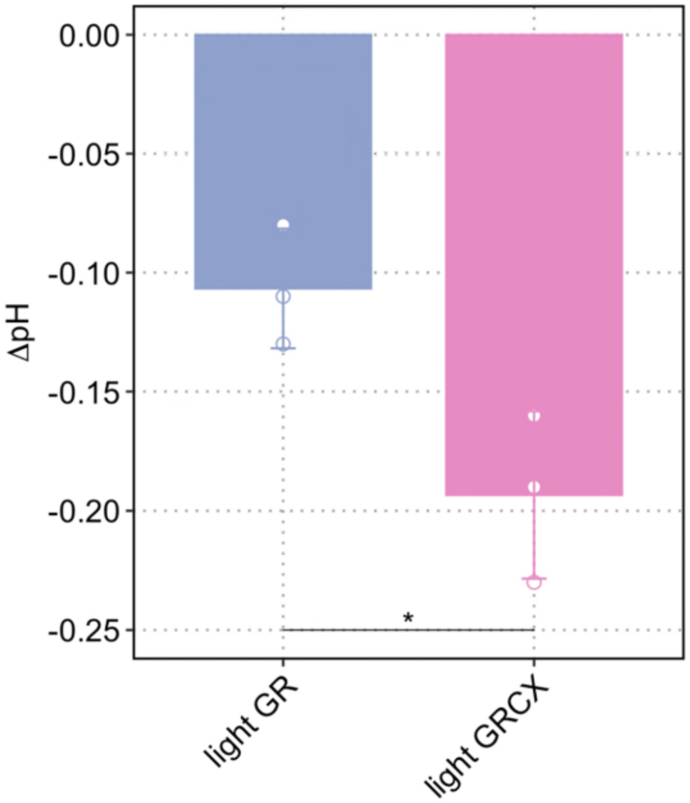

Supplementary Fig. 9. Extracellular proton concentration changes in cell suspensions with GR and GRCX over 1 min. Statistics were performed with Student's *t*-test (*: $p < 0.05$). Data are means ± SD of three biological replicates. Exact p value is 0.0298. Source data are provided as a Source Data file.

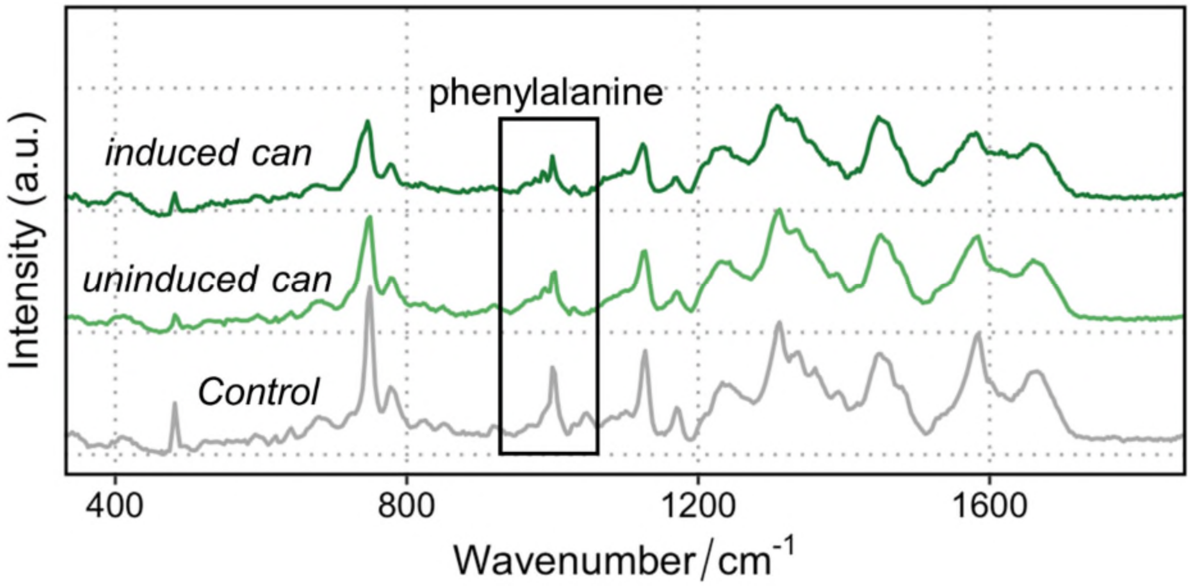

Supplementary Fig. 10. Averaged single-cell Raman spectra of induced and uninduced *R. eutropha* RHM5-GR-*can* cultured with 20 mM ^{13}C labelled bicarbonate, and phenylalanine is highlighted. Source data are provided as a Source Data file.

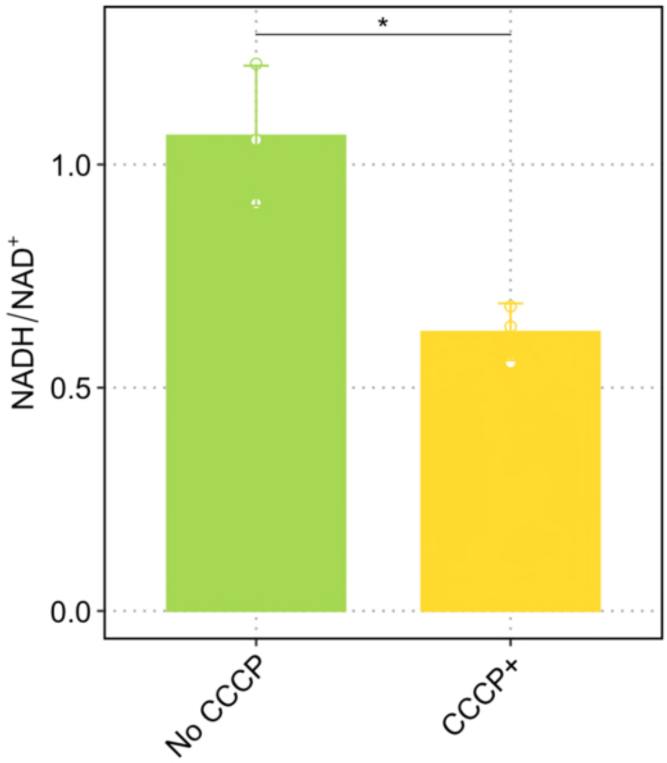

Supplementary Fig. 11. NADH/NAD$^+$ ratios in the *R. eutropha*-GR-Mtr under photoelectrosynthetic conditions with and without carbonyl cyanide m-chlorophenyl hydrazone (CCCP) treatment. Statistics were performed with Student's *t*-test (*: $p < 0.05$). Data are means ± SD of three biological replicates. Exact *p* value is 0.0267. Source data are provided as a Source Data file.

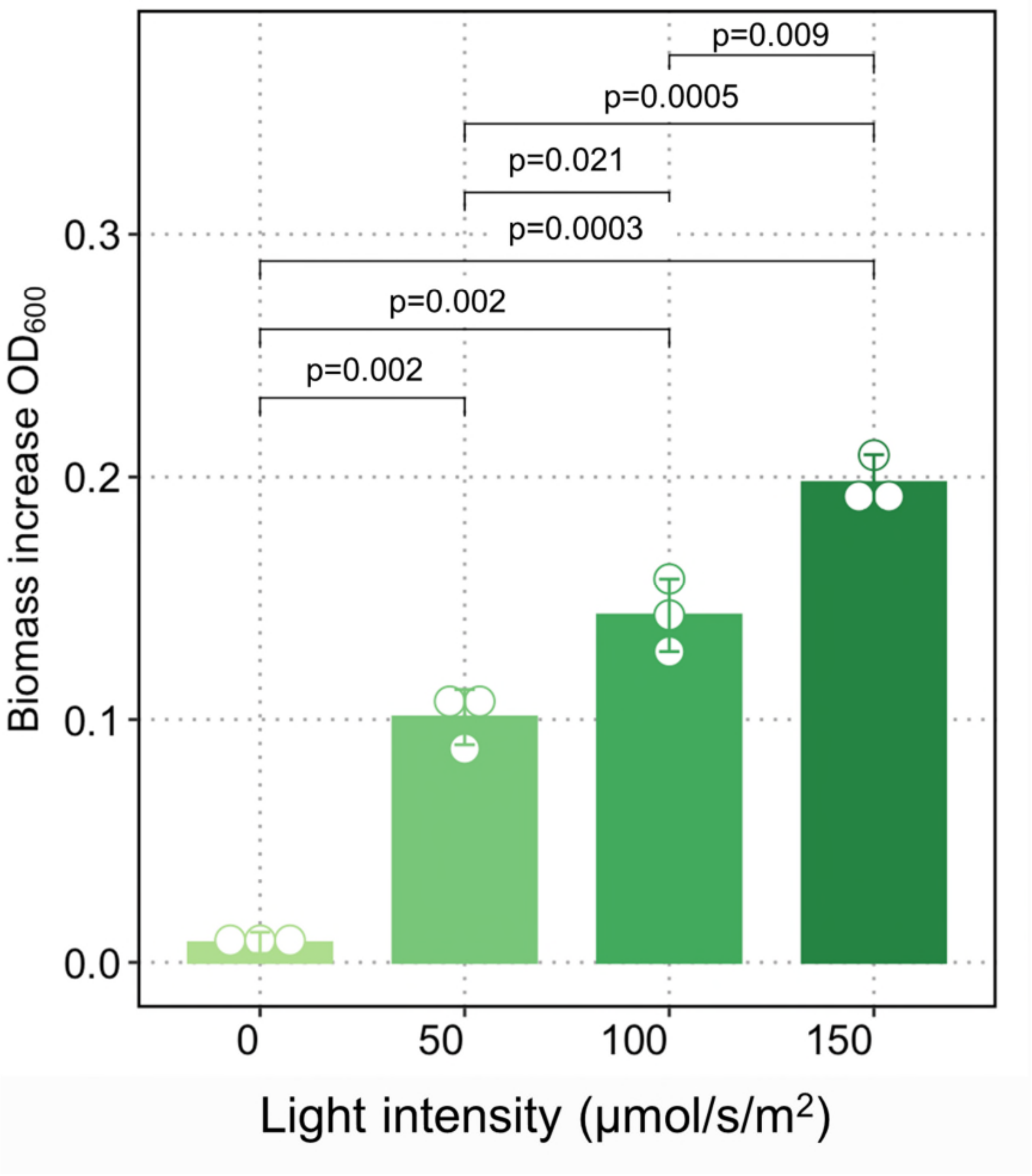

Supplementary Fig. 12. Biomass increases in *R. eutropha* RHM5-GR-Mtr calculated from changes in the OD_{600} over 5 days under different light intensities. Statistics were performed with Student's *t*-test. Data are means ± SD of three biological replicates. Exact *p* values are shown in the figure. Source data are provided as a Source Data file.

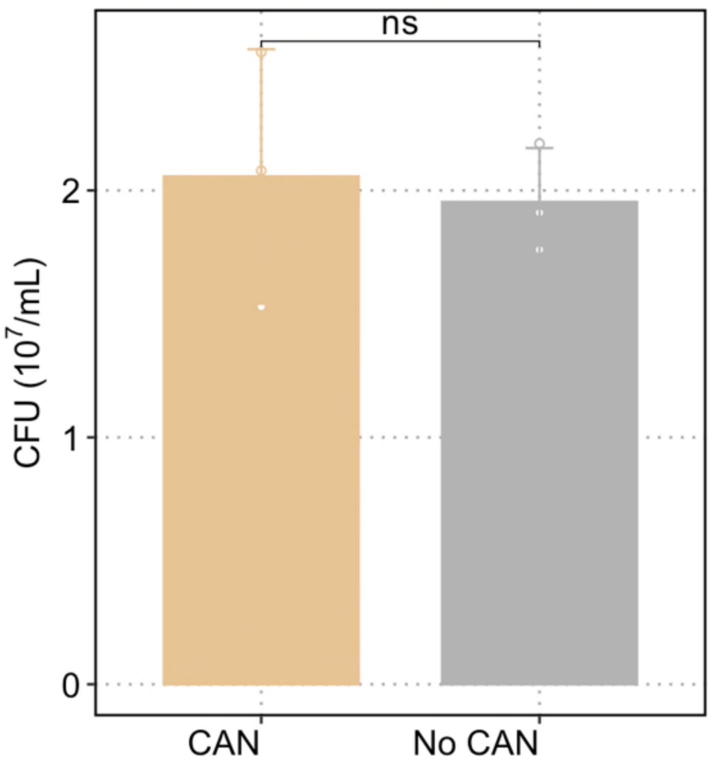

Supplementary Fig. 13. Comparison of the viability of the engineered cells with and without canthaxanthin after 3-day incubation under illumination. Statistics were performed with Student's *t*-test (ns: not significant). Data are means ± SD of three biological replicates. Exact *p* values are 0.772. Source data are provided as a Source Data file.

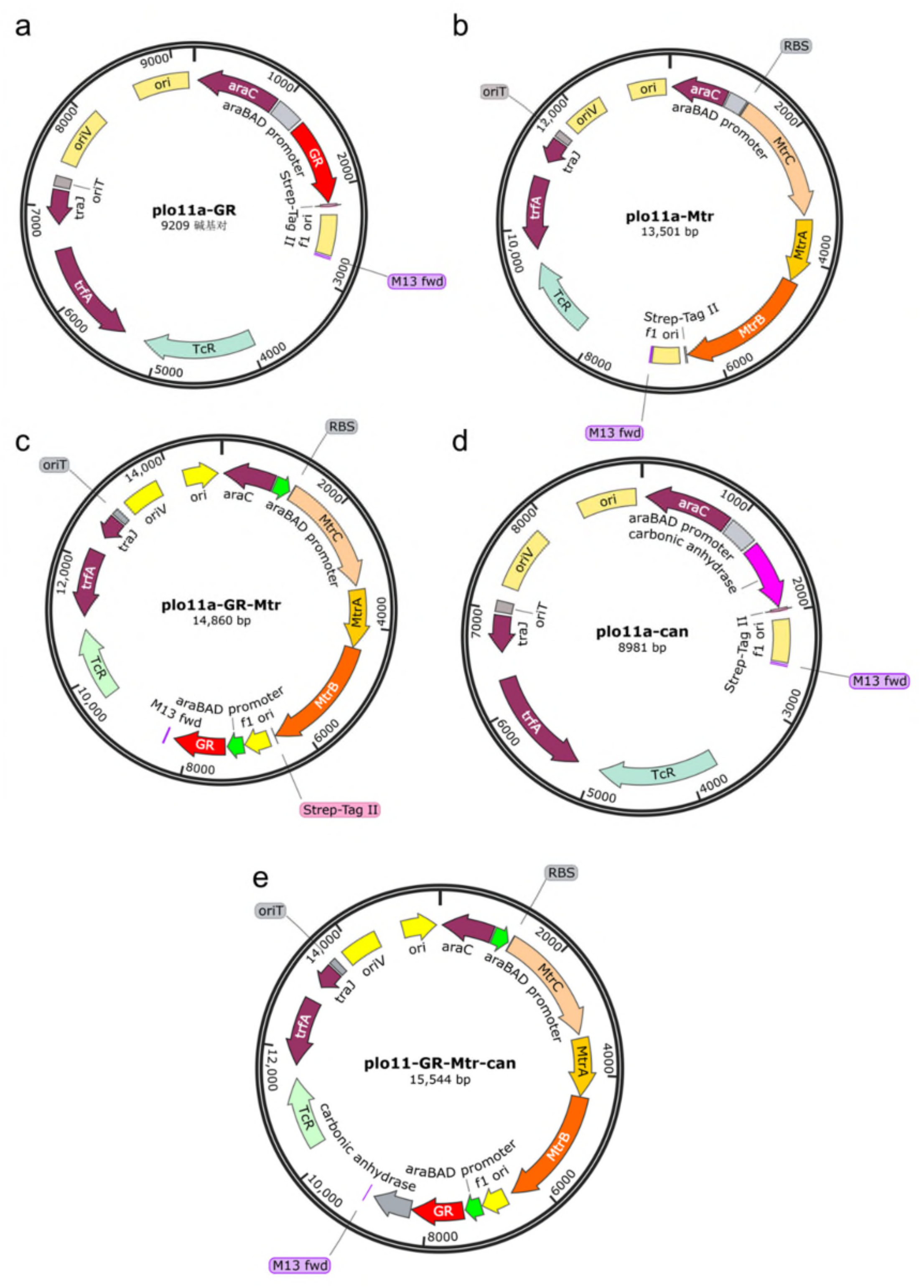

Supplementary Fig. 14. Plasmid maps. (a) pLO11a-GR, (b) pLO11a-Mtr, (c) pLO11a-GR-Mtr, (d) pLO11a-*can*, and (e) pLO11a-GR-Mtr-*can*.

Supplementary references

1. Thomas PE, Ryan D, Levin W. An improved staining procedure for the detection of the peroxidase activity of cytochrome P-450 on sodium dodecyl sulfate polyacrylamide gels. *Analytical Biochemistry* **75**, 168-176 (1976).

2. Jensen HM, *et al.* Engineering of a synthetic electron conduit in living cells. *Proceedings of the National Academy of Sciences* **107**, 19213-19218 (2010).

3. Davison PA, *et al.* Engineering a rhodopsin-based photo-electrosynthetic system in bacteria for CO_2 fixation. *ACS Synthetic Biology* **11**, 3805-3816 (2022).
